# New Progress of Epigenetic Biomarkers in Urological Cancer

**DOI:** 10.1155/2016/9864047

**Published:** 2016-08-09

**Authors:** Peng Wu, Ziyi Cao, Song Wu

**Affiliations:** ^1^Department of Urological Surgery, The Affiliated Luohu Hospital of Shenzhen University, Shenzhen University, Shenzhen 518000, China; ^2^Shenzhen Following Precision Medical Institute, Shenzhen Luohu Hospital Group, Shenzhen 518000, China; ^3^First Clinical Medical College, Anhui Medical University, Hefei 230032, China; ^4^Shenzhen Second People's Hospital, 1st Affiliated Hospital of Shenzhen University, Shenzhen 518037, China; ^5^Medical College, Shenzhen University, Shenzhen 518060, China; ^6^Shenzhen Gene Detection Public Service Platform of Clinical Application, Shenzhen Luohu Hospital Group, Shenzhen 518000, China

## Abstract

Urological cancers consist of bladder, kidney, prostate, and testis cancers and they are generally silenced at their early stage, which leads to the loss of the best opportunity for early diagnosis and treatment. Desired biomarkers are scarce for urological cancers and current biomarkers are lack of specificity and sensitivity. Epigenetic alterations are characteristic of nearly all kinds of human malignances including DNA methylation, histone modification, and miRNA regulation. Besides, the detection of these epigenetic conditions is easily accessible especially for urine, best target for monitoring the diseases of urinary system. Here, we summarize some new progress about epigenetic biomarkers in urological cancers, hoping to provide new thoughts for the diagnosis, treatment, and prognosis of urological cancers.

## 1. Introduction

Urological cancers are comprised of bladder, prostate, renal, and testis cancers, which are among the 10 most frequent cancers in man except testis cancer. So far, the gold standard diagnosis of urological cancer is pathological diagnosis and the early screening methods are rare. Some existing biomarkers such as prostate-specific antigen may be useful in prostate cancer screening but it is invasive and short of specificity and is causing overdiagnosis and overtreatment, which limits its application [1]. Bladder cancer and renal cell carcinoma lack specific predictive biomarkers and only some symptoms, for instance, hematuria, might have some effects in finding the existence of cancer. In the ear of personalized and precise medicine, searching noninvasive biomarkers to detect the presence of urological cancers early has become an urgent need. In this review, we focus on epigenetic-based biomarkers in urology system and summarize current state of research on epigenetic alterations [2], holding the promise to provide new ideas for the diagnosis, treatment, and prediction of urological cancer.

## 2. Epigenetic Mechanisms and Cancer

DNA methylation, mediated by DNA methyltransferases (DNMTs), using S-adenosyl methionine (SAM) as the methyl supplier and adding methyl group to the 5-carbon of the cytosine, is the most well delved epigenetic modification in human diseases [3, 4]. This phenomenon mostly occurs at the cytosine and guanine (CpG) dinucleotides [5], which dispersed in the genome or in DNA repetitive region. But, within the gene promoter regions, there are clusters of CpGs called CpG islands, usually remaining unmethylated to enable gene expression. DNA methylation and demethylation are known to be associated with tumorigenesis and result in silencing tumor suppressor genes and activating oncogenes, respectively [6]. Chemically speaking, DNA methylation is stable and can be accurately measured in almost all types of specimens, for instance, plasma, serum, and specimens [7]. Although DNA methylation is an ideal biomarker for clinical examination, there are still some obstacles to be overcome including DNA isolation efficiency, leukocyte DNA contamination, and loss of DNA templates arising from the employment of bisulfite treatment.

Histones modification includes methylation, carbonylation, phosphorylation, ubiquitinylation, sumoylation, glycosylation, and acetylation, which are known as “the histone code,” together with DNA methylation regulating the expression of specific genes [8]. The mechanism of regulation is through the addition of specific chemical groups to the N-terminal of basic amino acid residues at the tails of histones, altering the affinity of the histone tails to the DNA and changing the conformation of chromatin structure to regulate the transcription of genes. For instance, histone acetylation enhances the transcriptional activity of gene for lowering the affinity of histone tails to the DNA, while histone deacetylases result in gene repression [9, 10]. Contrary to the DNA methylation, the histone methylation (addition of methyl groups to H3, H4 lysine, and arginine) can generate two effects, either activation or repression transcription, depending on the specific amino acid residues modified [11]. In cancer cell, genome-wide histone modification and expression of key histone modulating enzymes have been reported, but the detailed mechanisms of their involvement in tumorigenesis are poorly understood [12, 13].

Another epigenetic modification has been studied deeply, known as microRNAs (miRNAs). miRNAs, a class of noncoding RNAs, which are about 18–25 nucleotides at length, could be synthesized and processed in the nucleus. miRNAs alter the expression through binding to mRNAs; then RNA-induced silencing complex takes part and regulates protein translation [14, 15]. It was estimated that at least 30% of human genes are regulated by miRNA. It is noteworthy that each mRNA can be combined with multiple miRNAs and each miRNA may regulate multiple mRNAs. miRNAs play an important role in tumorigenesis, activating oncogenes or restraining tumor suppressor genes [14, 15]. The ways that miRNAs alter gene expression include deletion, amplification, mutation, and chromosomal abnormalities [16]. Some studies found that miRNA can influence gene expression via targeting a specific gene region for DNA methylation and histone modifications [16, 17] and the expression of miRNA can be regulated by other epigenetic modifications such as DNA methylation [18, 19], which indicated the interactions between miRNAs and other epigenetic mechanisms.

## 3. Epigenetic Biomarkers in Bladder Cancer

Bladder cancer is the most common male urology malignancy in China and the total number of cases in males for 2015 is approximately 62.1 thousand [20]. Currently, the diagnosis of bladder is mainly invasive causing discomfort to patients and can only provide a generalized outcome for patients, so the demand for noninvasive screening and diagnosis method is urgent. Epigenetic biomarkers can decrease the use of invasive methods and provide the early diagnosis of bladder cancer allowing for more effective treatment. For example, RUNX3 gene that has been thought as tumor suppressor gene exhibited significant increase at methylation levels in bladder cancer by analyzing 124 tumor tissue samples. This study shows that RUNX3 gene may be a potential marker for detecting bladder cancer [21]. Apart from diagnostic markers, a prognostic indicator in patients with non-muscle-invasive bladder cancer (NMIBC) was found by Yoon et al. The clinical relevance of RSPH9 was determined by quantitative pyrosequencing analysis of 136 human bladder specimens (8 normal controls and 128 NMIBCs). They concluded that RSPH9 methylation can be of value for the assessment of disease recurrence and an independent prognostic indicator in NMIBC patients [22]. Similarly, Lin and Luo et al. reported that the hypermethylation of PCDH10 (50%, *n* = 117) and PCDH17 (52%, *n* = 151) was related to the development of bladder cancer and it was an independent predictor of cancer-specific survival time [23, 24].

With the deepening of research, it was thought that there was no single gene found to be methylated in vast majority of bladder tumors. More and more researches have focused on using gene panels to early detect bladder cancer and predict tumor recurrence, progression, and metastasis. A methylation analysis of PCDH17 and POU4F2 was developed recently in 148 individuals by qMSP using urine sediment. The combination of POU4F2/PCDH17 detection had sensitivity and specificity of 90.00% and 93.96% in the validation of 312 individuals [25]. Renard et al. found that 2 genes (TWIST1 and NID2) were frequently methylated in urine samples from bladder cancer patients (93% specificity and 90% sensitivity) [26]. Another study discovered that a methylation gene panel which consisted of 4 genes (CDH1, CDH13, RASSF1A, and APC) had significant correlations with poor prognosis (cancer with high grade, advanced stages, and aneuploidies). Likewise, a study conducted by Yates et al. demonstrated that promoter methylation of RASSF1A, CDH1, TNFSR25, EDNRB, and APC was associated with tumor progression [27].

Histone modifications were also found to relate to the pathogenesis of bladder cancer and regulation of cancer cell proliferation. A study in 2011 identified global histone H4K20 trimethylation that could predict cancer-specific survival in patients with muscle-invasive bladder cancer [28]. A meta-analysis published by An et al. showed that merged odds ratio of the effect between slow acetylation and bladder cancer was 1.31 (95% confidence odds ratio interval = 1.11–1.55), illustrating that slow acetylation modestly increases the risk of bladder cancer [29]. Recently, Jia et al. found that the acetylation status of KLF4 can decide the gene function as a tumor suppressor or oncogene in bladder cancer. Their results indicated that deacetylated KLF4 can act as an oncogene accelerating bladder cancer proliferation; on the contrary, acetylation of KLF4 performs as a tumor suppressor, which may be a good target for bladder cancer therapy [30]. In addition, another study found that expression levels of H3K4me1, H4K20me1, H4K20me2, and H4K20me3 were correlated with advanced pathological stage and that H4K20me3 was an independent prognosis factor for the survival of patients with muscle-invasive bladder cancer [31].

miRNAs are synthesized and processed in the nucleus and released into cytoplasm, binding to specific mRNA and inducing gene silence [32]. Recently, Jiang et al. demonstrated a four-miRNA panel (miR-422a-3p, miR-486-3p, miR-103a-3p, and miR-27a-3p) that can be used for muscle-invasive bladder cancer (MIBC) prediction with an area under the receiver operating characteristic curve (AUC) of 0.894 (95% CI: 0.864–0.931) by MiSeq sequencing on serum from 207 MIBC patients, 285 non-muscle-invasive bladder cancer (NMIBC) patients, and 193 controls [33]. Sasaki et al. found that miRNA-146a-5p is increased in bladder patients and decreased in patients after transurethral resection. What is more, levels of miRNA-146a-5p in urine are significantly higher in patients with high-grade tumors compared with those with low-grade tumors. Their results suggested that urinary miRNA-146a-5p might be useful as a new noninvasive diagnostic marker, therapeutic target, or anticancer agent [34]. A similar study conducted by Matsushita et al. shows that miRNA-145 (miR-145-5p, guide-strand, and miR-145-3p, passenger-strand) plays pivotal roles in bladder cancer (BC) cells by regulating UHRF1, which is overexpressed in BC clinical specimens. Further assay confirms that ectopic expression of either miR-145-5p or miR-145-3p in BC cells obviously suppressed cancer cell growth [35]. Findings on epigenetic biomarkers in bladder cancer are summarized in Table 1.

## 4. Epigenetic Biomarkers in Kidney Cancer

Kidney cancer is the third most common urology malignancy in China and there were about 66.8 thousand people diagnosed with renal carcinoma in 2015. Among these patients, 43.2 thousand are males and females only account for 35% [20]. Currently, there are no widely accepted tumor markers for clinical diagnosis of renal cell carcinoma. The clinical diagnosis of renal cell carcinoma mainly depends on imaging examination and the definite diagnosis is confirmed by pathological examination. Hauser et al. demonstrated that Wnt antagonist family genes can serve as biomarkers for diagnosis, staging, and prognosis in kidney cancer using tumor and serum DNA. They utilized methylation-specific PCR detecting the level of genes panels comprised of sFRP-1, sFRP-2, sFRP-4, sFRP-5, Wif-1, and Dkk-3 in 62 RCC samples and corresponding normal renal tissue. The results indicated that Wnt antagonist family genes detection had sensitivity of 79.0% and specificity of 75.8% and the serum DNA was significantly correlated with tumor grade and stage [36]. Besides, other studies have reported that some genes are highly specific for RCC patients in the level of DNA hypermethylation including VHL (91%) and RASSF1A (93%) [37]. Recently, another similar outcome showed that two genes, SMPD3 and FBXW10, are hypermethylated in ccRCC tissue samples compared with paired normal tissues. Interestingly, after 5-aza-2′-deoxycytidine treatment, mRNA expression of SMPD3 and FBXW10 was significantly upregulated indicating that these two genes can be a target for treatment and provide predictive information for clinical decisions [38]. Furthermore, a tumor suppressive gene, DAB2IP, reported by Wang et al. suggests that DAB2IP CpG1 methylation is a practical and repeatable biomarker for ccRCC, which can provide prognostic value that complements the current staging system. This research team validated the relation between CpG methylation biomarker (DAB2IP CpG1) and poor overall survival in TCGA (a cohort of 318 ccRCC patients) by pyrosequencing quantitative methylation assay in 224 ccRCC patients from multiple Chinese centers (MCHC set) and 239 patients from University of Texas Southwestern Medical Center [39].

Histone modifications in kidney cancer were found to have significant relevance with the prognosis of renal cell carcinoma (RCC). Patients with positive immunostaining of H3K4me2 and H3K18Ac but accounting for a lower proportion generally had a shorter 1-year survival probability than those with more histone modifications [40]. This study reveals that histone modification is changed in the progress of kidney cancer. Currently, there is no curative treatment for advanced renal cancer. Enhancing histone acetylation is a promising epigenetic-based therapy for cancer. Seligson et al. found that the combination treatment of ritonavir and panobinostat can enhance histone acetylation and inhibit renal cancer growth by suppressing the expression of histone deacetylase (HDAC). They tested this combination in murine subcutaneous xenograft model using Caki-1 cells and found that after a 10-day treatment tumor growth was inhibited significantly [41]. Furthermore, in a study with 21 clear cell renal cell carcinoma (ccRCC) patients, overexpression of CA9 was detected in all patients. Meanwhile, the expression levels of hMOF gene (an acetyltransferase) frequently were downregulated in 19 patients accompanied by the acetylation of histone H4K16. The study concluded that hMOF may be involved in the pathogenesis of kidney cancer and can be a new CA9-independent RCC diagnostic marker [42].

miRNAs are also involved in the development and progression of renal cell carcinoma. Zhang et al. reported that miRNA-126 can inhibit tumor cell invasion and metastasis by downregulating ROCK1 in renal cell carcinoma. They analyzed 128 pairs of ccRCC and adjacent normal tissue samples, measured miRNA-126 expression levels, and found the association between miRNA-126 and various clinicopathological parameters [43]. Using only one kind of miRNA marker might be inaccurate for prognosis; an analogous study concerning ccRCC presented that three miRNAs (miR-146a-5p, miR-128a-3p, and miR-17-5p) were correlated with metastasis of ccRCC patients. Specifically, they showed that the targeted genes were downregulated by miR-146a-5p and validated the interaction in cell culture experiments [44].

Findings on epigenetic biomarkers in kidney cancer are summarized in Table 2.

## 5. Epigenetic Biomarkers in Prostate Cancer

Screening of individuals without any cardinal symptom by the PSA test has been the focus of increasing criticism, primarily due to potential overtreatment and less comprehensive evaluation [45]. Candidate biomarkers will be separated with groups as molecular class, soluble proteins DNA methylation, mRNA, microRNA, and so forth [46–48].

Methylation levels of two genes, PCDH17 and TCF21, were quantified in a total of 12 cancer cell lines and 318 clinical samples. These two gene methylation levels provided a sensitivity rate of 96% for prostate cancer. The high exposing of PCDH17 and TCF21 methylation in prostate cancer cell lines was significantly different from primary tumor tissues. Furthermore, methylation levels were meaningfully lower in bladder and prostate nontumorous tissues, providing a biological influence as cancer biomarkers [49, 50]. In addition, diagnostic coverage might be improved by using gene panels including GSTP1/ARF/CDNK2A/MGMT and GSTP1/APC/RARB2/RASSF1A for urine and GSTP1/PTGS2/RPRM/TIG1 for serum samples [46].

It has been noticed that HOXB13 became overexpressed during malignant progression of the prostatic tissue and played an important role in the pathogenesis of the prostate gland as a novel biomarker for the prognosis of prostate cancer [51]. ADAM19 (a disintegrin and metalloproteinase 19) is a transmembrane and soluble protein concerned in cell phenotype through cell adhesion and proteolysis. It has been shown in special immunohistochemical studies that ADAM19 protein levels were more expressed compared to normal prostate tissue during prostate cancer biopsies [52]. As some reports said that the expression of SFRP1 inversely correlates with the Gleason score, survival rate and response for endocrine therapy expression are a favorable predictive and prognostic biomarker [53]. In other methods, PSF1 is expressed in high-grade prostate cancer and may also be a useful biomarker to identify patients for diagnosis [54]. Engrailed-2 (EN2) protein, a homeodomain-containing transcription factor expressed in prostate cancer and secreted into the urine, showed a highly specific and sensitive effect as a kind of biomarker for prostate cancer [55]. There are many proteins which obtain the same function like downregulated protein SLC18A2 [56] and unregulated protein TRPM4 in prostate cancer [57]. SOX2 was consistently downregulated, except in cell clusters lying within lymph node- (LN-) positive prostate cancer [58]. Some other special genes could be found to predict recurrence as XPO6 [59] and FMOD as biomarker for prostate cancer [45].

Other promising RNA markers are the transcripts of fusion genes which between the androgen-regulated transmembrane protease serine 2 gene (TMPSS2) and ERG transcription factors through chromosomal rearrangements become stable and produce a viable mRNA during transcription. TMPSS2-ERG transcription factors have been identified as promising urinary novel biomarkers [60].

Several potential microRNAs for prostate cancer have been identified as important biomarkers. Circulating miRNA-410-5p level was significantly higher in the prostate cancer patients than in normal patients [61]. Recently, a present study compared miR-18a expression with the peripheral blood of patients, benign prostatic hyperplasia (BPH) of patients, and normal individuals to evaluate the possibility of achieving noninvasive diagnosis for prostate cancer. In summary, the present results indicate that miR-18a expression is significantly higher in peripheral blood of patients with prostate cancer compared with two others. Peripheral blood oncogenic miR-18a may serve as a potential noninvasive biomarker for prostate cancer tissues, acting as an oncogenic miRNA [62]. On the other hand, miRNA-129 is a novel independent prognosic factor because of being downregulated significantly in prostate cancer. On the contrary, overexpression of miR-129 could develop tumor suppressive functions and prevent prostate cancer growth [63].

The curiousness of CTCs for specific tumor characteristics has drawn major attention over the past few years. The genomic profiles of CTCs have been found to be largely comparable to primary tumors and/or metastatic tissue, indicating that CTCs are able to reflect tumor characteristics including the extent of intratumoral and biological heterogeneity. CTCs have been shown to be tumorigenic and capable of forming new metastases [64–67]. Androgen receptor splice variant-7 (AR-V7), in circulating tumor cells (CTCs) from metastatic castrate-resistant prostate cancer (mCRPC) patients, received enzalutamide or abiraterone. The result shocked us as none of the 18 patients with detectable AR-V7 in CTCs had prostate-specific antigen (PSA) responses. Further, the median time to PSA progression after enzalutamide or abiraterone treatment was only 1.3-1.4 months in AR-V7-positive patients as compared to 5.3–6.1 months in AR-V7-negative patients. AR-V7 in CTCs was also associated with shorter survival [68].

In 1960, prostate-specific antigen (PSA) was first discovered by Rubin Flocks. Since the mid-80s, PSA has been the most commonly used biomarker for prostate cancer to judge current and future risk, detect response to treatments, and detect recurrence in all stages of the disease. Due to this work, the Food and Drug Administration (FDA), in USA, approved the use of PSA for monitoring recurrence after treatment. It was later known that it was human species-specific [69]. Prostate-specific antigen (PSA) testing may be used for PCA screening; however, significant problems regarding specificity, overdiagnosis, and overtreatment limit the acceptance of this marker [70]. Recently, macrophage inhibitory cytokine-1 (MIC-1) concentration along with the PSA assay could provide much improved specificity to the assay by a retrospective study. It is not difficult to see that MIC-1 concentration in serum was elevated in prostate cancer patients compared to normal and biopsy-negative individuals. What is more, the MIC-1 level was correlated with the progression of prostate cancer. The analysis based on MIC-1 and PSA concentrations in serum with the patient with prostate cancer status improved the specificity of the diagnosis without compromising the high sensitivity of the PSA test alone and has potential for the prognosis for patient therapy strategies [71]. Findings on epigenetic biomarkers in prostate cancer are summarized in Table 3.

## 6. Epigenetic Biomarkers in Testicular Cancer

Recent genomic studies have identified risk SNPs in testicular germ cell tumors (TGCT). Increased PDE11A, SPRY4, and BAK1 promoter methylation and decreased KITLG promoter methylation in familial TGCT cases versus healthy male family controls can be used to diagnose TGCT in the early time [72]. It is reported that LINE-1 methylation may be gender-specific, with a strong correlation between LINE-1 methylation levels associated with disease risk [73]. Besides that, if we knock down miR-199a-3p in a normal human testicular cell line (HT), this leads to elevation of DNMT3A2 (DNMT3A gene isoform 2) mRNA and protein levels. In clinical samples, DNMT3A2 was significantly overexpressed in malignant testicular tumor, which was inversely correlated with the expression of miR-199a-3p [74]. The methylation profile of cancer-associated genes in testicular cancer correlates with histological types and cancer-specific genes. Further methylation analysis in a larger cohort is needed to elucidate the role of genes role in testicular cancer development and potential for therapy, early detection, and disease monitoring [75].

## 7. Conclusions

In general, these findings provide new directions for detection, diagnosis, and prognosis of urological cancer which may revolutionize the clinical management of cancer patients. DNA methylation is the most explored modification in urological cancer and modifications in histone and miRNA are becoming a hot research topic. Nevertheless, the majority of these markers are single target discovered in a small amount of clinical cases and lack specificity; therefore a panel consisting of multiple targets is needed and this kind of research will become a trend. Besides, there is plethora of epigenetic biomarkers which has been identified, but only extremely rare biomarkers can be applied to clinical diagnosis and therapy targets. The selected biomarker has to be evaluated through rigid clinical trials in appropriate scale and determine the relevance between the biomarkers and clinical practice. We expect more novel publications about epigenetic biomarkers in urological malignances especially from large clinical cases.

## Figures and Tables

**Table 1:** Overview of bladder cancer biomarkers.

Biomarker	Sample	Type	Diagnosis, treatment, or prognosis
Bladder cancer			

RUNX3	Tissue	DNA methylation	Diagnosis
RSPH9	Urine	DNA methylation	Diagnosis, prognosis
PCDH10, PCDH17	Urine	DNA hypermethylation	Treatment, prognosis
PCDH17, POU4F2	Urine	DNA methylation	All
TWIST1, NID2	Urine	DNA methylation	Diagnosis
CDH1, CDH13, RASSF1A, APC	Urine	DNA methylation	Prognosis
RASSF1A, CDH1, TNFSR25, EDNRB, APC	Urine	DNA methylation	Prognosis
H4K20	Tissue	Histone modification	Prognosis
KLF4	Urine	Histone modification	Treatment
H4K20me3	Tissue	Expression level	Prognosis
miR-422a-3p miR-486-3p miR-103a-3p miR-27a-3p	Tissue (serum)	Overexpression	Prognosis
miRNA-146a-5p	Urine	Overexpression	Prognosis
miRNA-145	Urine	Overexpression	Prognosis

**Table 2:** Overview of kidney cancer biomarkers.

Biomarker	Sample	Type	Diagnosis, treatment, or prognosis
Kidney cancer			

Wnt family genes	Tissue (serum)	DNA methylation	Diagnosis, prognosis
VHL, RASSF1A	Tissue	DNA methylation	Diagnosis
SMPD3, FBXW10	Tissue	Hypermethylation	Diagnosis
DAB2IP	Tissue	Methylation	Prognosis
H3K4me2, H3K18Ac	Tissue	Histone modification	Prognosis
hMOF	Tissue	Histone modification	Diagnosis
HDAC	Tissue	Histone modification	Treatment
miRNA-126	Tissue	Downregulated	Treatment
miR-146a-5p miR-128a-3p miR-17-5p	Tissue	Downregulated	Prognosis

**Table 3:** Overview of prostate cancer biomarkers.

Biomarker	Sample	Type	Diagnosis, treatment, or prognosis
Prostate cancer			

PCDH17, TCF21	Tissue	DNA methylation	Diagnosis
GSTP1, ARF, CDNK2A, MGMT	Urine	DNA methylation	Diagnosis
GSTP1, APC, RARB2, RASSF1A	Urine	DNA methylation	Diagnosis
GSTP1, PTGS2, RPRM, TIG1	Tissue (serum)	DNA methylation	Diagnosis
HOXB13	Tissue	Overexpression	Prognosis
ADAM19	Tissue	Overexpression	Treatment
SFRP1	Tissue	Decreased expression	Diagnosis, prognosis
PSF1	Tissue	Overexpression	Diagnosis, prognosis
EN2	Tissue, urine	Overexpression	Diagnosis
SLC18A2	Tissue	Downregulated	Diagnosis
TRPM4	Tissue	Overexpression	Prognosis
SUX2	Tissue	Downregulated	Prognosis
XPO6	Tissue	Overexpression	Prognosis
